# Frequency and Overlap of Cachexia, Malnutrition, and Sarcopenia in Elderly Patients with Diabetes Mellitus: A Study Using AWGC, GLIM, and AWGS2019

**DOI:** 10.3390/nu16020236

**Published:** 2024-01-11

**Authors:** Satoshi Ida, Kanako Imataka, Shoki Morii, Keitaro Katsuki, Kazuya Murata

**Affiliations:** Department of Diabetes and Metabolism, Ise Red Cross Hospital, Ise 516-8512, Japan; k.ima.91.87@gmail.com (K.I.); v.p.s.0729@gmail.com (S.M.); ktaroktsuki@gmail.com (K.K.); healthcheck@ise.jrc.or.jp (K.M.)

**Keywords:** elderly patients with diabetes mellitus, cachexia, malnutrition, sarcopenia

## Abstract

This study aimed to estimate the frequency and overlap of cachexia, malnutrition, and sarcopenia in elderly patients with diabetes mellitus. Patients who were aged at least 65 years, had diabetes mellitus, and were regularly visiting the Ise Red Cross Hospital on an outpatient basis were included. The patients were assessed to determine whether they had cachexia, malnutrition, and sarcopenia according to the Asian Working Group for Cachexia criteria, the Global Leadership Initiative on Malnutrition criteria, and the Asian Working Group for Sarcopenia 2019 criteria. A total of 510 patients (310 men and 200 women) were analyzed in this study. Sarcopenia, cachexia, and malnutrition were found in 84 patients (16.4%), 40 patients (7.8%) (17.8% among patients with chronic diseases), and 110 patients (21.5%), respectively. Among patients with sarcopenia, the frequencies of cachexia and malnutrition were 30% and 71.4%, respectively. Among patients with cachexia, the frequencies of sarcopenia and malnutrition were 65% and 90%, respectively, and among those with malnutrition, the frequencies of sarcopenia and cachexia were 54% and 32.7%, respectively. The overlap among cachexia, malnutrition, and sarcopenia appears to be an important factor to be considered in the treatment of elderly patients with diabetes mellitus.

## 1. Introduction

Patients with diabetes mellitus are aging as the elderly population increases [1]. Elderly patients with diabetes mellitus are prone to decreased skeletal muscle mass, muscle weakness, and impaired physical function as effects of glucotoxicity, increased inflammation and oxidative stress, and diabetic neuropathy [2]. Therefore, muscle mass or function maintenance is important in elderly patients with diabetes mellitus [3]. Elderly patients with diabetes mellitus are also characterized by higher prevalence rates of comorbidities, such as chronic heart failure, chronic kidney disease, and malignant tumors, which can result in impaired appetite, weight loss, and reduced food intake. Sarcopenia or malnutrition is an important geriatric syndrome associated with diabetes mellitus in elderly people [4,5]. Sarcopenia is characterized by muscle weakness or declined physical function associated with decreased muscle mass due to aging, chronic inflammation, increased oxidative stress, decreased physical activities, and undernutrition [4]. Malnutrition, conversely, is a condition characterized by calorie and protein deficits related to a loss of appetite due to malabsorption, metabolic abnormality, or aging [5]. Furthermore, diagnostic criteria for cachexia, in which inflammation due to chronic diseases as well as malignant tumors is found as an underlying pathological condition, have been developed recently [6], and the importance of cachexia has attracted attention. Sarcopenia, malnutrition, and cachexia are associated with low activities of daily living, bone fractures, hospitalization, prolonged hospitalization, increased healthcare costs, and higher mortality rates [7,8,9,10]. Thus, early diagnosis of and early intervention for these conditions in elderly patients with diabetes mellitus are essential tasks.

Previous studies have reported varying frequencies of sarcopenia, malnutrition, and cachexia and different effects of these conditions on health outcomes, presumably because these conditions were defined differently from one study to another. The diagnostic criteria have been standardized to solve such problems. Specifically, the Asian Working Group for Sarcopenia (AWGS) 2019 criteria (based on decreased limb skeletal muscle mass, muscle weakness, and impaired physical function) [11] and the Global Leadership Initiative on Malnutrition (GLIM) criteria (including decreased weight or body mass index, decreased limb skeletal muscle mass, decreased food intake, chronic diseases, and increased c-reactive protein (CRP)) [12] have been developed for sarcopenia and malnutrition, respectively. Moreover, the diagnostic criteria for cachexia, which is a complex metabolic syndrome associated with chronic diseases as mentioned above, have been developed by the Asian Working Group for Cachexia (AWGC) (based on decreased body mass index, anorexia, increased CRP, and decreased grip strength) [6]. As mentioned above, sarcopenia, malnutrition, and cachexia have many similarities in definitions and are thus expected to overlap. In fact, previous studies in elderly people have reported overlap between these pathological conditions [13,14]. Meanwhile, as different treatments are required for sarcopenia, malnutrition, and cachexia, it is important to diagnose these conditions differently. Adequate nutrition is necessary to combat malnutrition, and a high level of physical activity, in addition to sufficient nutrition, is important for sarcopenia [15]. Drug therapy has recently become available for cachexia [16]. Therefore, in addition to single diagnoses of individual conditions, the possibility of overlap should be kept in mind to ensure that individual patients are diagnosed accurately.

To the best of our knowledge, no previous studies have investigated the overlap among sarcopenia, defined by the AWGS2019 criteria; malnutrition, defined by the GLIM criteria; and cachexia, defined by the AWGC criteria, in elderly patients with diabetes mellitus. As described above, many elderly patients with diabetes mellitus concurrently have chronic diseases and are treated mainly with diet therapy and exercise therapy. Therefore, sarcopenia, malnutrition, and cachexia are important factors to consider in designing treatment plans. This study aimed to estimate the overlap among sarcopenia as defined by the AWGS2019 criteria, malnutrition as defined by the GLIM criteria, and cachexia as defined by the AWGC criteria in elderly patients with diabetes mellitus.

## 2. Materials and Methods

### 2.1. Study Design and Subjects

This was a cross-sectional study in patients who were aged at least 65 years, had diabetes mellitus, and visited the Ise Red Cross Hospital in Ise-shi, Mie, on an outpatient basis. Among patients with diabetes mellitus aged ≥ 65 years who visited the outpatient clinic of our hospital between July 2019 and December 2022, those who had undergone assessments to determine whether they had chronic diseases (chronic heart failure, cancer, and chronic kidney failure), cachexia according to the AWGC criteria (body mass index (BMI), anorexia, CRP, and grip strength) [6], malnutrition according to the GLIM criteria (BMI, limb skeletal muscle mass, decreased food intake, chronic disease, and CRP) [12], and sarcopenia according to the AWGS2019 criteria (limb skeletal muscle mass, grip strength, and physical function) [11] were eligible. Patients with alcohol intoxication, those with severe mental illness, those residing in a nursing care facility, and those unable to cooperate independently in the survey were excluded. This study was conducted after patients provided written consent, and approval was obtained from the ethics committee of the Ise Red Cross Hospital.

### 2.2. Assessment of Chronic Diseases

Chronic diseases included in the AWGC and GLIM criteria were chronic heart failure, cancer, and chronic kidney failure, which are common comorbidities in elderly patients with diabetes mellitus. Information from medical interviews or medical records was used to determine whether patients had chronic diseases according to the following criteria: (1) chronic heart failure and cancer, diagnosis and treatment of these conditions by a cardiologist or a physician in charge of the treatment of cancer; (2) chronic kidney failure, with an estimated glomerular filtration rate of <30 mL/min/1.73 m^2^.

### 2.3. Assessment of Cachexia

Cachexia was diagnosed when a patient with a causal chronic disease had a BMI of <21 kg/m^2^ and at least one of anorexia, increased CRP, and decreased grip strength [6]. The Japanese version of the Simplified Nutritional Appetite Questionnaire (SNAQ) translated by Nakatsu et al. [17] was used to evaluate patients for anorexia. The SNAQ is a self-administered questionnaire composed of four questions about appetite. The total score ranges from 4 to 20 points, with a higher score indicating higher appetite. In this study, scores < 14 were considered to indicate decreased appetite based on previous studies. CRP levels (measured by latex agglutination) ≥ 0.5 mg/dL were considered elevated based on the AWGC criteria. A Smedley dynamometer (Grip-D, Takei Scientific Instruments, Co., Ltd., Niigata City, Japan) was used to measure grip strength, and the maximum values for the left and right hands were averaged. Grip strength < 28 kg for men and <18 kg for women was defined as decreased grip strength.

### 2.4. Assessment of Sarcopenia

Sarcopenia was diagnosed based on the AWGS2019 criteria [11]. Multi-frequency bioelectrical impedance analysis (seca medical body composition analyzers 525, GmbH & Co., Hamburg, Germany) was used to measure limb skeletal muscle mass. The appendicular skeletal muscle mass (ASM) values were measured, and the limb skeletal muscle mass index (ASMI) values were calculated as follows: ASMI (kg/m^2^) = ASM (kg)/(body height (m)^2^). ASMI values lower than 7.0 kg/m^2^ for men and 5.7 kg/m^2^ for women were defined as reduced limb skeletal muscle mass. Grip strength values were measured, and the maximum values for the left and right hands were averaged; averaged values < 28 kg for men and <18 kg for women was defined as muscle weakness. The five times sit-to-stand test was used to evaluate physical function. The length of time required to sit and stand five times in succession was measured using a stopwatch. Patients requiring 12 s or longer to sit and stand five times were considered to have impaired physical function. A patient with a decreased skeletal muscle mass and a decreased grip strength or impaired physical function was diagnosed with sarcopenia.

### 2.5. Assessment of Malnutrition

GLIM criteria comprise phenotypic and etiologic criteria, and those meeting at least one each of phenotypic criteria (weight loss, low BMI, and decreased ASMI) and etiologic criteria (insufficient food intake and inflammation/chronic diseases) were diagnosed with malnutrition [12]. Of the phenotypic criteria, weight loss could not be measured in this study; thus, only low BMI (BMI < 18.5 kg/m^2^ for those aged < 70 years and BMI < 20 kg/m^2^ for those aged ≥ 70 years) and decreased ASMI (<7.0 kg/m^2^ for men and <5.7 kg/m^2^ for women) were considered. The specific etiologic criteria were as follows: (1) decreased food intake in those who answered “yes” to the question “Has your food intake decreased recently?” and (2) inflammation/chronic diseases in those with chronic diseases (chronic heart failure, cancer, and chronic kidney failure) or CRP ≥ 0.5 mg/dL.

### 2.6. Assessment of Other Variables

The following information was collected: age, sex, smoking habit, alcohol use habit, exercise habit, etiologic classification of diabetes mellitus (type 1, type 2), duration of diabetes mellitus, hemoglobin A1c (HbA1c), hypertension, dyslipidemia, diabetic retinopathy, diabetic neuropathy, living alone, cognitive function decline, physical frailty, and medications for diabetes mellitus. For the etiologic classification of diabetes mellitus, the disease was classified into type 1, type 2, and others based on the diagnostic criteria of the Japan Diabetes Society [18]. HbA1c was measured with high-performance liquid chromatography based on the National Glycohemoglobin Standardization Program. Systolic and diastolic blood pressure values were measured in examination rooms, and patients who had a systolic blood pressure of ≥130 mmHg or a diastolic blood pressure of ≥80 mmHg or were undergoing treatment with an antihypertensive agent were considered to have hypertension. For lipid levels, a patient was considered to have dyslipidemia if triglycerides ≥150 mg/dL, high-density lipoprotein cholesterol <40 mg/dL, or low-density lipoprotein cholesterol (LDL-c) ≥120 mg/dL (for patients with coronary artery disease, LDL-c ≥100 mg/dL) or if the patient was taking an oral lipid-lowering agent. Diabetic retinopathy was diagnosed by an ophthalmologist. The condition was diagnosed based on decreased Achilles’ tendon reflexes, decreased vibration perception in the lateral malleolus, or abnormalities in nerve conduction studies. Patients who answered “no” to the following yes–no question were considered to have cognitive function decline: “Can you recall what happened 5 min ago?” The frailty screening index (FI) developed by Yamada et al. [19] was used to evaluate physical frailty. The FI is a 5-item self-administered questionnaire composed of yes–no questions about weight loss, walking speed, exercise habit, memory, and fatigue. The total score ranges from 0 to 5 points, and patients with a score of ≥3 were considered to have physically frailty.

### 2.7. Statistical Analysis

Patient background variables were described separately for chronic diseases, cachexia, malnutrition, and sarcopenia. The normality of continuous variables was evaluated using the Shapiro–Wilk test. For group comparisons, normally and non-normally distributed continuous variables were assessed using analysis of variance and the Kruskal–Wallis test, respectively. Binary variables were compared between groups using the chi-square test. Among subjects with cachexia, malnutrition, and sarcopenia, prevalence rates of other pathological conditions were also calculated to evaluate the size of overlap. The significance level (two-tailed) was <0.05, and STATA version 16.0 (Stata Corporation LP, College Station, TX, USA) was used for analysis.

## 3. Results

A total of 870 patients provided consent to participate in the study during the observation period. Of these patients, 510 (310 men and 200 women) for whom data from assessment of chronic diseases, cachexia, malnutrition, and sarcopenia were available were analyzed in this study. The patient backgrounds are shown in Table 1. The median age was 75 years; men accounted for 60%; the median duration of diabetes mellitus was 18 years; and the median HbA1c was 7.4%. With regard to chronic diseases (224 patients in total), 118 patients had chronic heart failure, 86 had cancer, and 60 had chronic kidney failure (there was overlap). The median BMI in the group of subjects with sarcopenia, cachexia, and malnutrition was lower than that in the group of subjects without these conditions, and patients with type 1 diabetes mellitus, diabetic retinopathy, and physical frailty and those not treated with oral hypoglycemic agents in the former group were higher than those in the latter group.

**Figure 1:**
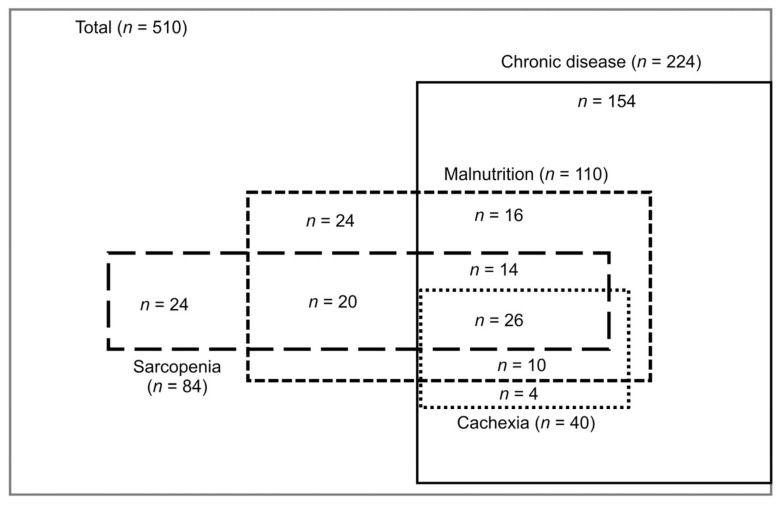
Overlap of sarcopenia (S – – ), cachexia (C ▪ ▪ ▪ ▪), and malnutrition (M - - - ).

Frequencies of chronic diseases, sarcopenia, cachexia, and malnutrition in those with the respective conditions are listed in Table 2 and illustrated in Figure 1. The frequencies of chronic diseases, sarcopenia, cachexia, and malnutrition were 224 patients (43.9%), 84 patients (16.4%), 40 patients (7.8%) (17.8% in patients with chronic diseases), and 110 patients (21.5%), respectively. The frequencies of chronic diseases, cachexia, and malnutrition in patients with sarcopenia were 47.6%, 30%, and 71.4%, respectively. The frequencies of chronic diseases, sarcopenia, and malnutrition in those with cachexia were 100%, 65%, and 90%, respectively. The frequencies of chronic diseases, sarcopenia, and cachexia in those with malnutrition were 60%, 54%, and 32.7%, respectively.

## 4. Discussion

The frequencies and overlap rates of cachexia according to the AWGC criteria, malnutrition according to the GLIM criteria, and sarcopenia according to the AWGS2019 criteria in elderly patients with diabetes mellitus were determined. Regarding overlap, the frequencies of sarcopenia and malnutrition in subjects with cachexia were extremely high, and the overlap between sarcopenia and malnutrition was also high. Meanwhile, the overlap rates of cachexia and malnutrition with chronic diseases were high, but the overlap rates of these conditions with sarcopenia were only approximately 50%. To the best of our knowledge, this is the first study to find overlapping patterns among these conditions in elderly patients with diabetes mellitus.

Virtually no reports investigating the frequency of cachexia in elderly patients with diabetes mellitus could be found, except for one reporting 9.4–12.8% [20]. In our study, the frequency of cachexia in elderly patients with diabetes mellitus and chronic diseases was 17.8%, which tended to be higher than the rate reported in the previous study. The possible reasons for the difference are as follows: In the previous study, cachexia was defined according to Evans et al. (BMI < 20 kg/m^2^ and at least three of muscle weakness, malaise, anorexia, decreased lean mass, and biochemical abnormalities) [20]. Compared with the AWGC criteria, the definition of Evans et al. uses a smaller BMI threshold and is expected to be met by a smaller number of individuals because at least three of muscle weakness, malaise, anorexia, decreased lean mass, and biochemical abnormalities are required. Another reason may be the effects of differences in patient groups between the two studies. Specifically, the previous study [20] included elderly patients with diabetes mellitus, regardless of whether they had chronic diseases. However, our study using the AWGC criteria only included patients with chronic diseases who were considered to have a high risk of cachexia. Presumably, the use of different definitions for cachexia and patient populations with different characteristics might have had effects on the difference in cachexia frequency between the two studies. In the present study, the frequency of sarcopenia in subjects with cachexia was as high as the rates in previous studies. The AWGC criteria include the decreased grip strength criterion in addition to low BMI, which is strongly associated with decreased limb skeletal muscle mass, an essential requirement for the diagnosis of sarcopenia [6]; this is a possible reason for the high frequency. Furthermore, the frequency of malnutrition in patients with cachexia in the present study was as high as 90%. The AWGC and GLIM criteria include a criterion of having a low BMI as an essential requirement and the presence of inflammation and chronic diseases; this may explain why the frequency of malnutrition was high.

A previous study in elderly diabetic patients with chronic kidney disease, which also used the GLIM criteria as in our study, reported that the frequency of malnutrition was 23.7% [21]. Previous studies in inpatients with diabetes mellitus [22,23] have reported that the frequency of malnutrition according to the GLIM criteria was 23.6–52.9%. Thus, the frequency of malnutrition in the present study in elderly outpatients with diabetes mellitus was similar to that in previous studies. In the present study, the frequency of sarcopenia in patients with malnutrition was 54%, meaning that approximately half of the patients with malnutrition had sarcopenia. A previous study in elderly nursing home residents hospitalized [14] reported that the frequency of sarcopenia in patients with malnutrition according to the GLIM criteria was 31.2%. Decreases in skeletal muscle mass, muscle mass, and physical function in patients with diabetes mellitus have been reported to be greater than those in non-diabetic patients [2]; this may explain why the frequency of sarcopenia was high in our study. Meanwhile, the GLIM phenotypic criteria include decreased limb skeletal muscle mass in addition to low BMI, which is strongly associated with decreased limb skeletal muscle mass. However, the GLIM etiologic criteria do not include grip strength and physical function, which are necessary to diagnose sarcopenia. Therefore, it was considered important to diagnose sarcopenia independently of malnutrition.

In previous studies in diabetic patients aged ≥ 65 years, the frequency of sarcopenia has been reported to range from 5.8% to 15.3% [24,25]. A study investigating frequencies of sarcopenia in different age groups [24] reported a frequency of 13.9% in those approximately 75 years of age, which is the mean age of participants in our study; rates in this study and ours are similar. Meanwhile, in the present study, the frequency of chronic diseases in subjects with sarcopenia was 47%, which seems to be lower than the rates in those with cachexia or malnutrition. Previous studies have shown that patients with chronic diseases, such as chronic heart failure and chronic kidney disease, had high frequencies of sarcopenia [26,27]. Moreover, as described above, patients with diabetes mellitus have been reported to have greater decreases in skeletal muscle mass, muscle mass, and physical function [2], and the importance of sarcopenia has been suggested. The results of the present study imply that the evaluation of sarcopenia is important in elderly diabetic patients with or without chronic diseases.

The results of this study revealed a significant overlap of cachexia, malnutrition, and sarcopenia and are of clinical significance in the following aspects: These three pathological conditions have many features in common, but importantly, they differ etiologically and thus require different measures. Specific measures important for the respective conditions include control of inflammation caused by chronic diseases for cachexia; monitoring of food intake and adjustment of nutritional balance for malnutrition; and increased physical activities, including resistance exercise as well as increased nutritional intake for sarcopenia [15]. In elderly patients with diabetes mellitus, measures against malnutrition are considered particularly important because their food intake has been restricted since they were middle-aged, without adequate measures to prevent malnutrition in some cases. Recently, drug therapy for cachexia has become available [16], and the diagnosis of this condition is important. The overlap of cachexia, malnutrition, and sarcopenia in elderly patients with diabetes mellitus is an important factor to consider in designing treatment strategies.

This study is the first to evaluate the overlap of sarcopenia, malnutrition, and cachexia in elderly patients with diabetes mellitus, which is the strength of this study. Because these conditions require different treatments, the study’s results are useful for establishing treatment strategies by considering the overlap among these conditions. Furthermore, because sarcopenia, malnutrition, and cachexia in this study were defined based on the AWGS 2019 criteria, GLIM criteria, and AWGC criteria, respectively, the use of these internationally standardized criteria allowed the results of this study to be compared with those of other studies, which is another strength of this study.

This study has some limitations. First, the participants in this study were patients visiting an outpatient clinic specialized for diabetes mellitus, and many had relatively severe diabetes mellitus. Therefore, care should be exercised in determining whether the findings of this study can be applied to patients with glycemic stability or those visiting their primary care physicians. Second, the sample size in this study was relatively small, and further analysis in a larger number of patients is desirable. Third, chronic diseases analyzed in this study did not include chronic obstructive lung disease and collagenosis, such as rheumatism. Therefore, the frequency of cachexia might have been underestimated and should be interpreted with caution. Fourth, the accuracy in estimating the prevalence of malnutrition may be influenced as our study did not assess weight loss, one of the three phenotypic criteria as defined by the GLIM guidelines. Fifth, the impact of medications like glucagon-like peptide-1 (GLP-1) receptor agonists and sodium-glucose cotransporter-2 (SGLT-2) inhibitors on food intake and body weight was not considered. These drugs are known to reduce appetite and body weight, potentially leading to an overestimation of malnutrition within our findings. Sixth, ASM values were evaluated after being adjusted by height. Thus, the results of this study may differ if these values were assessed after being adjusted by other factors, such as body weight and BMI [28]. Further research is required to compensate for the above limitations.

## 5. Conclusions

Despite the limitations described above, this study showed the frequency and overlap of cachexia according to the AWGC criteria, malnutrition according to the GLIM criteria, and sarcopenia according to the AWGS2019 criteria in elderly patients with diabetes mellitus. This study indicated that cachexia patients have a high frequency of sarcopenia and malnutrition, and sarcopenia and malnutrition highly overlap. Furthermore, while the overlap rates of cachexia and malnutrition with chronic diseases were high, the overlap rates of these conditions with sarcopenia were approximately half of those. It is clinically important to consider the overlap among sarcopenia, malnutrition, and cachexia when establishing the treatment strategy for elderly patients with diabetes mellitus who are suffering from these conditions.

## Figures and Tables

**Table 1:** Characteristics of the analysis population.

	Total *n* = 510	Chronic Disease *n* = 224 (43.9%)	Sarcopenia *n* = 84 (16.4%)	Cachexia *n* = 40 (7.8%)	Malnutrition *n* = 110 (21.5%)
Components of sarcopenia, cachexia, or malnutrition					
Low ASMI, %	25.8	25.0	100 *	56.4 *	81.8 *
Low grip strength, %	40.7	46.4 *	85.7 *	64.1 *	54.5 *
Low physical performance, %	32.5	35.7	52.3 *	33.3	36.3
Etiology, %	43.9	100	47.6	51.2	60.0 *
Low BMI (<21 kg/m^2^), %	20.7	19.6	42.8 *	100 *	54.5 *
Low appetite, %	23.0	26.4	17.1	31.4	27.0
Elevated CRP, %	27.8	27.6	26.1	51.2 *	43.6 *
Reduced food intake, %	24.3	31.2 *	35.7 *	41.0 *	43.6 *
Age (years), median (range)	75.0 (72.0–79.0)	76.0 (72.0–81.0) *	76.5 (71.5–80.0)	79.0 (75.0–82.0) *	77.0 (73.0–82.0) *
Male, %	60.7	67.8	40.4 *	58.9	58.1
BMI (kg/m^2^), median (range)	23.7 (21.4–26.2)	24.3 (21.5–26.8) *	21.1 (18.6–23.4) *	19.1 (18.3–20.1) *	20.8 (19.1–22.0) *
T2DM, %	90.2	95.5 *	78.5 *	85.0 *	78.1 *
Duration of diabetes (years), median (range)	18.0 (12.0–25.0)	18.0 (12.0–24.0)	20.0 (14.0–29.0)	18.0 (12.0–29.5)	17.5 (12.0–25.0)
HbA1c (%), median (range)	7.4 (6.7–7.9)	7.3 (6.6–7.7) *	7.5 (6.8–8.5) *	7.3 (6.7–7.7)	7.4 (6.7–7.9)
Alcohol consumption, %	15.2	14.2	7.1 *	7.6	10.9
Smoking, %	14.1	16.9	14.2	15.3	14.5
Hypertension, %	76.7	84.8 *	80.9	66.6	74.5
Dyslipidemia, %	69.8	76.7 *	73.8	55.2	63.6
Retinopathy, %	37.6	44.6 *	57.1 *	41.0 *	47.2 *
Neuropathy, %	36.8	40.1	50.0 *	46.1	38.1
Cardiovascular disease, %	21.5	50.4 *	25.0	27.0	27.2 *
Living alone, %	13.3	12.5	11.9	17.9	18.1
Cognitive function decline, %	20.0	20.0	28.9 *	29.6	25.0
Physical frailty, %	26.0	33.3 *	43.2 *	47.3 *	43.5 *
Oral hypoglycemic agents, %	75.6	74.1	64.2 *	53.8 *	65.4 *
Sodium-glucose cotransporter 2 inhibitors, %	16.4	13.8	5.7 *	4.5 *	7.1 *
Insulin, %	69.6	73.8	76.1	64.1	76.3
GLP-1RA, %	19.2	25.0 *	16.6	17.9	20.0

BMI, body mass index; T2DM, type 2 diabetes mellitus; HbA1c, hemoglobin A1c; GLP-1RA, glucagon-like peptide-1 receptor agonist. * Significant difference between patients with and without the respective condition (*p* < 0.05).

**Table 2:** Prevalence of each within chronic disease, sarcopenia, cachexia, and malnutrition.

	Chronic Disease *n* = 224	Sarcopenia *n* = 84	Cachexia *n* = 40	Malnutrition *n* = 110
Chronic disease, %	–	47.6	100	60.0
Sarcopenia, %	17.8	–	65.0	54.5
Cachexia, %	17.8	30.9	–	32.7
Malnutrition, %	29.4	71.4	90.0	–

## Data Availability

The data presented in this study are available on request from the corresponding author. The data are not publicly available due to ethical.
